# Anesthetic management of a morbidly obese patient with endometrial cancer during robot-assisted laparoscopic surgery

**DOI:** 10.1186/s40981-021-00434-y

**Published:** 2021-04-05

**Authors:** Yusuke Ishida, Koichi Nakazawa, Toshio Okada, Yumi Tsuzuki, Takayuki Kobayashi, Rikako Yamada, Hiroyuki Uchino

**Affiliations:** Department of Anesthesiology, Tokyo Medical University, 6-7-1 Nishishinjuku, Shinjuku-ku, Tokyo, 160-0023 Japan

**Keywords:** Severe obesity, Robot-assisted laparoscopic surgery, Endometrial cancer

## Abstract

**Background:**

The number of robot-assisted surgeries being performed has increased in recent years, even in patients with risk factors, such as obesity, owing to advancements in medical technologies. We here report the anesthetic management of a morbidly obese woman who underwent robot-assisted surgery.

**Case presentation:**

A 44-year-old woman (height, 165 cm; weight, 147 kg; body mass index, 54 kg/m^2^) was scheduled to undergo robot-assisted laparoscopic hysterectomy for endometrial cancer. Preoperative weight loss and rehearsal of positioning during induction of anesthesia and surgical procedures greatly contributed to the surgical success. Monitoring of oxygen reserve index in combination with SpO_2_ was useful for appropriate airway and respiratory management. During anesthesia induction, the ramp position using a special commercially available cushion facilitated manual mask ventilation and tracheal intubation. Lung-protective ventilation using a limited tidal volume with moderate PEEP was applied during the robot-assisted surgical procedure.

**Conclusion:**

We successfully managed anesthesia without any complications.

## Background

The da Vinci robotic system has been found to be highly useful for procedures in narrow body cavities, such as for pelvic surgery. We here report the anesthetic management of a morbidly obese patient with a body mass index (BMI) of more than 50 kg/m^2^, who underwent robot-assisted hysterectomy. We obtained written informed consent from the patient to publish this case report.

## Case presentation

**Fig. 1:**
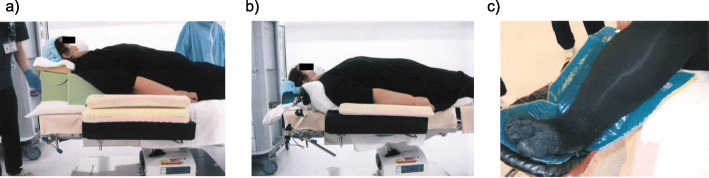
Simulation of anesthesia and surgery with the patient. The team of anesthesiologists, surgeons, and nurses who were involved in the surgery confirmed whether the patient could get on the bed, whether the bed could then be moved, whether the optimal posture for securing the airway during anesthesia could be adopted, and whether any other preparations for intraoperative contingencies were required. **a** The position of the patient during induction of anesthesia. A ramp pillow was used for placing the patient in the position for improving upper airway patency. **b** Supine position after tracheal intubation. **c** The lower extremities in the lithotomy position

A 44-year-old woman (height, 165 cm; weight, 147 kg; BMI, 54 kg/m^2^) who was diagnosed with endometrial cancer was scheduled for a robot-assisted hysterectomy under general anesthesia. We instructed the patient to lose weight before the operation. In addition, 2 weeks before the surgery, we assessed the patient in the operating room in the positions that would be required during anesthesia induction, and in the supine, lithotomy, and Trendelenburg positions (Fig. 1).

At the time of admission, the patient’s body weight had decreased to 137 kg due to a balanced diet. Her blood pressure, heart rate, and peripheral blood oxygen saturation (SpO_2_) while breathing room air were 110/70 mmHg, 74 bpm, and 97%, respectively. Maquet Meera OR Table (Getinge AB, Getinge, Sweden), which can withstand a patient weight of up to 250 kg regardless of position, was used for the surgical table.

**Fig. 2:**
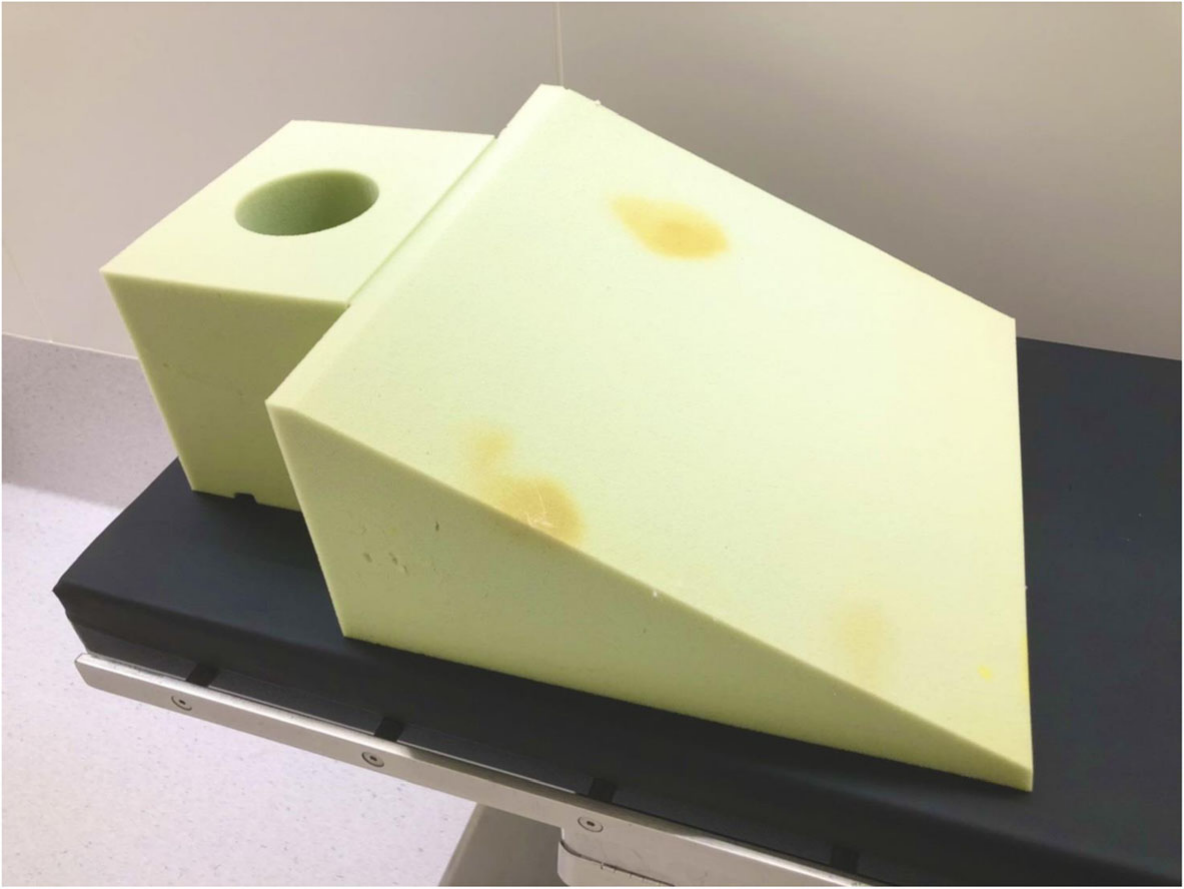
The ramp pillow used for anesthesia induction and tracheal intubation. The Pi’s pillow was used for placing the patient in the ramp position at the time of anesthesia induction and tracheal intubation. The Pi’s pillow consists of a base and a removable pad (pillow), which are designed for maintaining appropriate positioning for mask ventilation, laryngoscopy, and tracheal intubation, particularly for obese patients

**Fig. 3:**
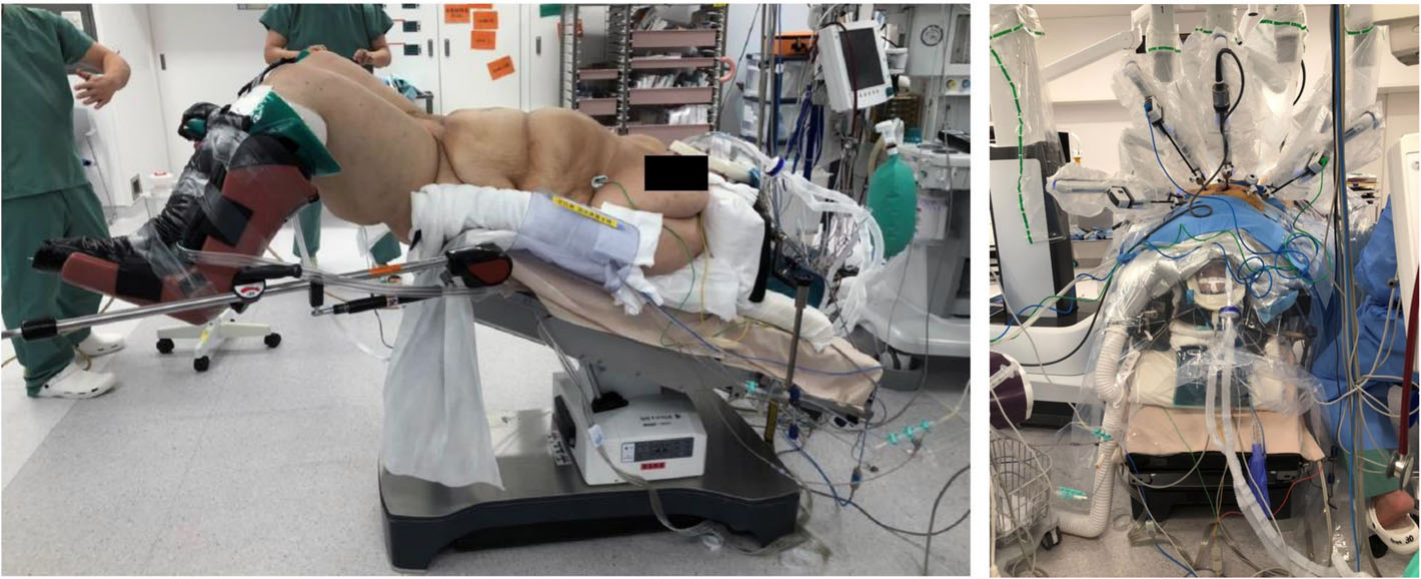
Intraoperative position of the patient. The patient was placed in the head-down position both before the surgery (left) and intraoperatively (right)

**Fig. 4:**
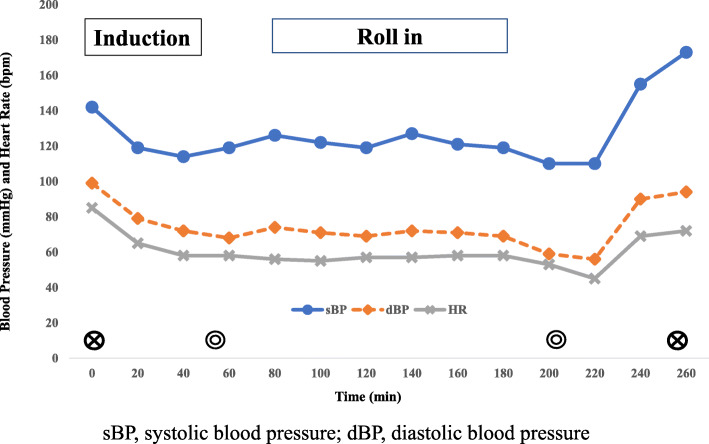
Anesthetic record. sBP, systolic blood pressure; dBP, diastolic blood pressure

During the induction of anesthesia, a Pi’s pillow (American Eagle Medical LLC, Uniondale, NY, USA) was used to maintain the patient in the ramp position (Fig. 2), and she was preoxygenated via a facemask using an oxygen flow of 6 L/min. The Oxygen Reserve Index (ORi^TM^; software version updated since January 2018 in Japan) (Masimo Co., Irvine, CA, USA) in combination with SpO_2_ was used to evaluate oxygenation during anesthesia. After confirming an increase in ORi to 0.52, anesthesia was induced with remifentanil (0.3 μg/kg/min: actual body weight) and propofol (200 mg), and muscle relaxation was achieved with rocuronium bromide (90 mg) under neuromuscular monitoring (TOF-Cuff ®; RGB Medical Devices, Madrid, Spain). The trachea was intubated with an 8.0 mmID endotracheal tube using a McGrath MAC® video laryngoscope (Medtronic Co., Minneapolis, MN, USA). A blade size of 4 was used. Anesthesia was maintained with 2% sevoflurane, the continuous infusion of remifentanil (0.1–0.2 μg/kg/min), and rocuronium bromide, and sedation levels were monitored using the patient state index (PSI) measured with a Sedline® monitor (Masimo Co., Irvine, CA, USA). PSI levels were maintained at scores between 30 and 50. Intraoperatively, intermittent pneumatic compression of the lower extremities was performed to prevent deep vein thrombosis. Before commencing robotic surgery, the patient was placed in a steep Trendelenburg lithotomy position (30° head down), and pneumoperitoneum was created with CO_2_ insufflation at a pressure of approximately 10 mmHg (Fig. 3). Mechanical ventilation was performed by pressure control ventilation (FiO_2_, 0.45; inspiratory pressure, 30 cmH_2_O; PEEP, 10 cmH_2_O; and inspiratory to expiratory time ratio, 1:2), and the respiratory rate was adjusted to maintain end-tidal CO_2_ at between 40 and 50 mmHg. Although SpO_2_ and ORi were used as references for oxygenation, SpO_2_ fluctuated between 96% and 98% with ORi of 0.00 during the robot-assisted procedure, suggesting no oxygen reserve for maintaining SpO_2_. ORi increased transiently in response to lung recruitment maneuvers by manual inflation to a 40 cmH_2_O airway pressure. After returning the patient to the supine position and the cessation of pneumoperitoneum, ORi increased to 0.17 under a FiO_2_ of 0.45. The surgery was completed without any substantial hemodynamic changes. For postoperative analgesia, fentanyl was intravenously administered to achieve a target blood concentration of 0.5 ng/mL, followed by a 0.5 μg/kg/h intravenous patient-controlled analgesia infusion. Before emergence from anesthesia, the cuff leak test was performed to ensure a difference in expired tidal volume with and without cuff inflation. We confirmed a significant amount of air leakage (>150 ml) with cuff deflation and ruled out airway edema. Neuromuscular blockade was reversed with sugammadex (4 mg/kg), and the patient was extubated after confirmation of complete neuromuscular recovery by the train-of-four ratio. As the patient’s circulatory and respiratory dynamics were stable, she was returned to the ward. The operating time was 156 min and duration in the Trendelenburg position was 100 min (Fig. 4). During the first 12 h postoperatively, the patient was receiving 5 L/min of oxygen, with SpO_2_ of 99–100% and a respiratory rate of 16 in the ward. The patient was able to ambulate from the day after surgery, and she was discharged from the hospital without any complications 7 days postoperatively.

## Discussion

There are several factors to consider in the management of morbidly obese patients undergoing robot-assisted gynecological surgeries. Preoperative assessment of the patient’s chronic medical conditions, such as hypertension, cardiovascular disease, central or obstructive sleep apnea, and impaired glucose tolerance, is indispensable. Preoperatively, evaluation and preparation for the management of a difficult airway should be performed. During robotic surgery of obese patients with pneumoperitoneum using CO_2_ insufflation and a steep Trendelenburg position, the decrease in chest wall compliance is augmented by the large amount of fatty tissue in the thoracoabdominal region, which increases the risk of atelectasis and hypercapnic acidosis. Protective lung strategies using low tidal volumes or limiting plateau airway pressure and PEEP can be challenging in these circumstances. Furthermore, increases in intrathoracic pressure might subsequently increase intraocular and intracranial pressures [1–4], and induce upper airway edema [5]. Not only are these intraoperative problems increased in morbidly obese patients, but pharmacological considerations are also more complicated.

For the present patient, preoperative evaluation and management were crucial for successful perioperative management. As we received a preoperative consultation from the surgeon in charge 1 month prior to the surgery, we were able to instruct the patient to lose weight before the surgery, and she successfully lost 10 kg. In addition, we simulated the surgery in the operating room with the patient before the surgery, to confirm that there were no inconveniences or flaws regarding the procedure for establishment of the airway and when the patient was in the Trendelenburg position. The patient’s SpO_2_ did not decrease below 90% during the actual operation in this position.

In obese patients, desflurane, which has a low blood gas partition coefficient, confers greater intraoperative control of the depth of anesthesia, as well as rapid and consistent postoperative emergence and recovery [6]. However, in the present case, the patient had experienced an asthma attack 2 months before the surgery. Thus, sevoflurane, which causes less airway irritability and a relatively strong bronchodilation effect, was considered to be more preferable.

The dosing of rocuronium in obese patients is still controversial [7, 8]. Rocuronium is a slightly lipophilic drug, and it has been suggested that the duration of action of such a drug is prolonged when administered based on actual body weight rather than ideal body weight. However, there are reports suggesting that at a rocuronium dose of 0.6 mg/kg, there is no difference in the recovery rate even when it is administered according to the patients’ actual weight [9]. We acknowledge that administering a drug based on the patient’s ideal body weight is safer for morbidly obese patients, considering their postsurgical recovery. In the present patient, a dose of 0.6 mg/kg of her actual weight was administered to facilitate tracheal intubation, followed by continuous infusion under neuromuscular monitoring to maintain the train-of-four ratio at 0 and post-tetanic count between 0 and 5 [10–12]. After surgery, 4 mg/kg of sugammadex was sufficient to achieve full recovery of neuromuscular activity, as confirmed by a train-of-four ratio of 1.0. Extubation was performed without any sequelae, and no pulmonary complications were observed postoperatively.

For postoperative analgesia, we simulated fentanyl blood concentrations by pharmacokinetic simulations using the Shafer model [13]. But it may be inappropriate in an extremely obese patient. We should have used Shibutani’s “pharmacokinetic mass” for our simulation. Using “pharmacokinetic mass” and the time course of fentanyl administration, we will be able to estimate fentanyl effect-site concentrations more accurately [14, 15].

Placing a patient with pneumoperitoneum in the Trendelenburg position promotes atelectasis, particularly in morbidly obese patients [16]. Although PEEP and recruitment maneuvers are effective for the prevention of atelectasis, a reasonable approach to determining the appropriate level of PEEP has not yet been established. The PEEP level of 10 cmH_2_O that was used in the present patient appeared to be insufficient, because during the surgery, the SpO_2_ fluctuated between 96 and 98% with an ORi of 0.00, and SpO_2_ increased to 100% following the recruitment maneuver whereas ORi spontaneously increased to 0.30. Atelectasis thus appeared to develop at a PEEP of 10 cmH_2_O. Mazzinari et al. measured esophageal pressure in patients who underwent laparoscopic cholecystectomy, and showed that by setting the PEEP to a value 2 cmH_2_O higher than the intraperitoneal pressure, the driving pressure based on transpulmonary pressure can be reduced compared with that using the standard PEEP value (5 cmH_2_O) [17]. In the present case, as the patient’s intraperitoneal pressure was 10 mmHg (≒ 13 cmH_2_O) and she was placed in the Trendelenburg position, it might have been more appropriate if the PEEP level had been set to more than 15 cmH_2_O. Furthermore, as pressure-controlled ventilation was set to achieve a tidal volume of 7 mL/kg based on the patient’s ideal weight, the plateau pressure was approximately 29 cmH_2_O and the driving pressure was 19 cmH_2_O. Therefore, in the present patient, the PEEP level should have been set to a higher value while lowering the plateau pressure. ORi was monitored throughout the anesthetic period to keep track of oxygenation. ORi is an index reflecting moderate hyperoxia (PaO_2_ of 100–200 mmHg) and is expressed on a non-unit scale of 0.00 to 1.00. During the induction of anesthesia, ORi increased to 0.52 owing to preoxygenation, and from after intubation to after the patient was positioned in the Trendelenburg position, ORi fluctuated between 0.20 and 0.30, with an FiO_2_ of 0.45. We believe that ORi is a useful reference for the estimation of oxygenation, and that the measurement of ORi enabled us to perform early interventions, such as the setting of PEEP levels and performing recruitment maneuvers [18–20].

Obesity exacerbates the complications associated with robot-assisted pelvic surgery. Specifically, proper airway and respiratory management is difficult when obese patients are placed in the Trendelenburg position. Therefore, rehearsal of the surgery in the Trendelenburg position prior to the actual surgery should be performed, and in addition, appropriate respiratory management and evaluation of oxygenation using ORi are also very helpful. Anesthetics, such as remifentanil, rocuronium, and sugammadex, which facilitate the awakening and recovery of obese patients and reduce postoperative lung complications, should be used. It is well known that robot-assisted laparoscopic surgery reduces surgical site infections and shortens the duration of hospitalization; hence, in the future, this type of surgery is expected to become more common, even in obese patients.

## Data Availability

Not applicable

## Abbreviations

BMI: Body mass index
SpO_2_: Peripheral blood oxygen saturation
PEEP: Positive end-expiratory pressure
ORi: Oxygen Reserve Index (ORi^TM^)
CO_2_: Carbon dioxide
FiO_2_: Fraction of inspired oxygen
PaO_2_: Arterial partial pressure of oxygen
